# Rolling Bearing Fault Diagnosis Based on VMD-MPE and PSO-SVM

**DOI:** 10.3390/e23060762

**Published:** 2021-06-16

**Authors:** Maoyou Ye, Xiaoan Yan, Minping Jia

**Affiliations:** 1 School of Mechatronics Engineering, Nanjing Forestry University, Nanjing 210037, China; yemaoyou@njfu.edu.cn; 2 School of Mechanical Engineering, Southeast University, Nanjing 211189, China; mpjia@seu.edu.cn

**Keywords:** variational modal decomposition, multiscale permutation entropy, particle swarm optimization-based support vector machine, rolling bearing, fault diagnosis

## Abstract

The goal of the paper is to present a solution to improve the fault detection accuracy of rolling bearings. The method is based on variational mode decomposition (VMD), multiscale permutation entropy (MPE) and the particle swarm optimization-based support vector machine (PSO-SVM). Firstly, the original bearing vibration signal is decomposed into several intrinsic mode functions (IMF) by using the VMD method, and the feature energy ratio (FER) criterion is introduced to reconstruct the bearing vibration signal. Secondly, the multiscale permutation entropy of the reconstructed signal is calculated to construct multidimensional feature vectors. Finally, the constructed multidimensional feature vector is fed into the PSO-SVM classification model for automatic identification of different fault patterns of the rolling bearing. Two experimental cases are adopted to validate the effectiveness of the proposed method. Experimental results show that the proposed method can achieve a higher identification accuracy compared with some similar available methods (e.g., variational mode decomposition-based multiscale sample entropy (VMD-MSE), variational mode decomposition-based multiscale fuzzy entropy (VMD-MFE), empirical mode decomposition-based multiscale permutation entropy (EMD-MPE) and wavelet transform-based multiscale permutation entropy (WT-MPE)).

## 1. Introduction

With the vigorous development of the machinery industry, rolling bearings have become an important component of rotating machinery and are widely used in generators, gas engines and other kinds of rotating machinery [1,2]. The local fault of rolling bearing will directly affect the normal operation of the whole of the mechanical equipment, so it is important to explore a new fault diagnosis technique. However, in practical engineering, the collected bearing vibration signals contain various interference signals (e.g., white noise, harmonic interference) and have nonlinear and nonstationary properties, which indicates that it is difficult to distinguish the bearing fault types and severities. Therefore, to improve the fault diagnosis accuracy of rolling bearings, it is very necessary to remove the useless information and obtain more accurate fault features by a novel fault diagnosis method [3,4,5].

Common vibration signal decomposition methods include empirical mode decomposition (EMD) [6], wavelet transform (WT) [7], variational mode decomposition (VMD) [8,9], etc. EMD has been widely addressed by many scholars and is applied in the field of bearing fault diagnosis. Sun et al. [10] proposed a fast bearing fault diagnosis method based on ensemble empirical mode decomposition (EEMD), the moth-flame optimization algorithm based on Lévy flight (LMFO) and the naive bayes (NB). The results manifest the efficiency and accuracy of signal sparse representation, and fault type classification has been enhanced. However, EMD has some disadvantages, such as end effect and mode aliasing [11]. In addition, in the decomposition process of EMD, its performance depends heavily on extreme point search and envelope interpolation. WT is an effective time-frequency analysis method in signal decomposition, but its performance largely depends on the selection of wavelet basis function. That is, WT is not adaptive in signal decomposition. To overcome the deficiencies of EMD and WT, Dragomiretskiy [12] proposed a new adaptive time-frequency analysis method named VMD in 2014. Compared with EMD and WT, VMD can suppress interference signals, avoid the loss of useful information and provide high-quality data sources for subsequent feature extraction. In this method, the center frequency and bandwidth of each mode are determined automatically by iteratively searching the optimal solution of variational modes. That is, VMD has the characteristics of high decomposition accuracy and high operational efficiency, which can effectively suppress the phenomenon of mode aliasing in the process of signal decomposition. At present, it has been applied to many fields, such as rotating machinery fault diagnosis, by many scholars. For instance, Li et al. [13] firstly adopted whale optimization algorithm-based optimized VMD to decompose lidar signals into several intrinsic mode functions (IMF); then, based on Bhattacharyya distance criterion, the relevant modes are selected to reconstruct the original signal and complete the effective feature extraction. Li et al. [14] firstly used VMD to obtain a series of intrinsic mode functions (IMF), then selected the IMF with a higher center frequency as the main IMF component and generalized the compound multiscale symbolic dynamic entropy (GCMSDE) of the main IMF component, which is calculated for fault feature extraction and planetary gearbox fault diagnosis. However, in the above studies, only one or several main mode components obtained by VMD are adopted for fault feature information extraction, which may cause a loss of some useful fault information of the original bearing vibration signal. Therefore, considering the advantages of VMD, this paper adopts the VMD method to decompose the collected bearing vibration signal. Furthermore, to avoid some shortcomings of the existing studies, after conducting VMD, the feature energy ratio (FER) criterion is introduced to reconstruct the bearing vibration signal in this paper, which can not only adequately retain the useful fault characteristic information but also remove some interference frequency components.

To analyze the feature information of the mode components obtained from VMD decomposition, Shannon entropy theory is introduced. Permutation entropy (PE) [15], as a measurement tool of time series complexity, is highly sensitive to signal mutation and has made good progress in the field of fault diagnosis. However, PE cannot measure multiscale signals. It has a great disadvantage in bearing fault feature extraction. Therefore, multiscale permutation entropy (MPE) [16,17] is introduced into fault diagnosis. MPE not only contains the characteristics of a simple calculation and strong anti-interference of PE but can also be used to analyze the signal at different scales. Du et al. [18] used MPE to extract fault features and, combined with the self-organizing fuzzy classifier based on harmonic mean difference (HMDSOF), to classify fault features. The results verified the superiority of MPE. In view of the limitations of existing fault diagnosis methods for rotating machinery in single-scale signal analysis, Li et al. [19] proposed a fault diagnosis method based on MPE and a multichannel fusion convolutional neural network (MCFCNN) and verified that this method has high diagnostic accuracy, stability and speed. Compared with PE, MPE is more stable and can be used in a wider range. Therefore, this paper adopts the MPE method to extract bearing fault features.

Common classification and identification methods include the artificial neural network (ANN) [20], extreme learning machine (ELM) [21,22] and support vector machine (SVM) [23,24]. Although ANN has obtained many achievements in the field of pattern recognition, its identification performance greatly depends on its several important parameters (e.g., the number of layers and nodes). Additionally, ANN can easily fall into the local minimum value in the optimization process. Although ELM runs fast, its generalization performance is poor. SVM has fewer adjustable parameters and runs stably. It can obtain higher diagnostic accuracy under the condition of fewer training samples. Mao et al. [25] adopted SVM to classify and identify the transformer winding type correctly. Compared with ANN and ELM, SVM has the advantages of simple calculation, stable operation, good robustness and better global optimization performance. Moreover, the identification performance of SVM is greatly affected by its several important parameters. Hence, this paper uses the particle swarm optimization (PSO) to automatically determine the important parameters of SVM and adopts the parameter-optimized SVM to identify bearing fault types. To summarize, the main work and contributions of this paper are summarized as follows:

(1) The VMD method based on the feature energy ratio (FER) criterion is presented to decompose and reconstruct the original bearing vibration signal, which can retain the abundant bearing fault information and remove some interference components.
(2) The VMD, MPE and PSO-SVM are combined into an effective fault diagnosis method, which can improve the identification accuracy of bearing faults.
(3) Two experimental cases are conducted to show the effectiveness of the proposed method in bearing fault identification.

The rest of this paper is organized as follows. Section 2 introduces the basic theory of some methods (i.e., VMD, MPE, PSO-SVM). Section 3 shows the flowchart of the proposed bearing fault diagnosis method. In Section 4, the effectiveness of the proposed method is proven by using two experimental examples. Furthermore, contrastive analysis among the different methods is conducted in Section 4. Finally, some conclusions are summarized in Section 5.

## 2. Related Algorithm

### 2.1. Variational Mode Decomposition

The purpose of the VMD is to decompose the original signal f into several mode components $u_{k}$ [26]. $\omega_{k}$ represents the center frequency of each mode component. $u_{k}$ is defined as the amplitude modulation (AM) and frequency modulation (FM) signal, and the expression is as follows:

(1) $$u_{k}(t)=A_{k}(t)cos\left(\varphi_{k}\left(t\right)\right)$$

where $A_{k}(t)$ is the instantaneous amplitude of the signal, and $\varphi_{k}(t)$ is the instantaneous phase of the signal.

The frequency center and bandwidth of each mode function are determined by the extreme value of the variational mode constructed by the iterative search, which realizes the frequency domain division of the signal and the effective separation of each component. The constrained variational model is firstly constructed as follows:

(2) $$\begin{array}{l} \underset{\left\{u_{k}\},\{\omega_{k}\right\}}{min}\left\{\sum_{k}{\left\|\partial t\left[\left(\delta (t)+\frac{j}{\pi t}\right)u_{k}(t)\right]e^{-j\omega_{k}t}\right\|}_{2}^{2}\right\}\\ s.t.\sum_{k}u_{k}=f \end{array}$$

where $\left\{u_{k}\right\}=\left\{u_{1},\ldots ,u_{k}\right\}$ and $\left\{\omega_{k}\right\}=\left\{\omega_{1},\ldots ,\omega_{k}\right\}$ are modal functions and center frequencies, respectively.

The quadratic penalty factor α and Lagrange multiplication operator λ are introduced to establish the Lagrange function:

(3) $$\begin{array}{l} L(\{u_{k}\},\{\omega_{k}\},\{\lambda_{k}\})=\alpha {\sum_{k}\left\|\partial t\left[\left(\delta (t)+\frac{j}{\pi t}\right)u_{k}(t)\right]e^{-j\omega_{k}k}\right\|}_{2}^{2}+\\ {\left\|f(t)-\sum_{k}u_{k}(t)\right\|}_{2}^{2}+\langle \lambda (t),f(t)-\sum_{k}u_{k}(t)\rangle \end{array}$$

where f(t) represents the original input signal.

Transform Equation (3) from time-domain to frequency-domain by Fourier transform, and calculate the corresponding extreme value. Finally, the corresponding mode components $u_{k}$ and $\omega_{k}$ are obtained:

(4) $${\hat{u}}_{k}^{n+1}\left(\omega \right)=\frac{\hat{f}\left(\omega \right)-\sum_{i\neq k}{\hat{u}}_{i}\left(\omega \right)+\frac{\hat{\lambda }\left(\omega \right)}{2}}{1+2\alpha {\left(\omega -\omega_{k}\right)}^{2}}$$

(5) $$\omega_{k}^{n+1}=\frac{\int_{0}^{\infty }\omega {\left|{\hat{u}}_{k}\left(\omega \right)\right|}^{2}d\omega }{\int_{0}^{\infty }{\left|{\hat{u}}_{k}\left(\omega \right)\right|}^{2}d\omega }$$

where $u_{k}$ is the *k*-th mode component obtained by signal decomposition, and $\omega_{k}$ is the center frequency corresponding to the *k*-th mode component.

### 2.2. Signal Reconstruction Based on Feature Energy Ratio Criterion

This paper adopts the reconstruction method based on the feature energy ratio criterion to reconstruct the IMF component of VMD decomposition. This method can not only make full use of the IMF information but can also retain the useful fault characteristic information and remove some interference frequency components. Specific steps of signal reconstruction based on feature energy ratio criterion are as follows:

Firstly, the feature energy ratio $FER_{k}$ of each mode component is calculated:

(6) $$FER_{k}=\left(E_{k}^{1}+E_{k}^{2}+\ldots +E_{k}^{h}\right)/E_{k}$$

where $E_{k}^{h}$ is the accumulated energy of the feature frequency at the *h* order in the Hilbert envelope spectrum of the *k*-th mode component $u_{k}$, and $E_{k}$ is the total energy of the envelope spectrum of the *k*-th mode component $u_{k}$. Briefly speaking, $E_{k}^{h}$ is obtained based on Equation (4). More specifically, suppose that one given signal x(t) is decomposed by VMD to obtain the *k*-th mode component $u_{k}$. The envelope spectrum of $u_{k}$ is calculated by Equation (7). $E_{k}^{h}$ is the amplitude energy corresponding to the *k*-th fault frequency of $S_{k}(f)$:

(7) $$\begin{array}{l} s_{k}(t)=\left|u_{k}(t)+j\cdot Hilbert[u_{k}(t)]\right|\\ S_{k}(f)=DFT[s_{k}(t)] \end{array}$$

where $s_{k}(t)$ is the envelope signal of the *k*-th mode component $u_{k}(t)$, DFT[·] denotes the Fourier transform operator and $S_{k}(f)$ represents the envelope spectrum of the *k*-th mode component $u_{k}(t)$.

Secondly, the reconstruction weight $\beta_{k}$ of each mode component and the normalized reconstruction weight ${\hat{\beta }}_{k}$ are calculated:

(8) $$\beta_{k}=\frac{FER_{k}}{\sum_{k=1}^{k}FER_{k}}$$

(9) $${\hat{\beta }}_{k}=\frac{\beta_{k}}{\max(\beta )}$$

Finally, the reconstructed signal $x_{F\mathrm{inal}}$ is obtained:

(10) $$x_{Final}={\hat{\beta }}_{1}x_{1}+{\hat{\beta }}_{2}x_{2}+\ldots +{\hat{\beta }}_{k}x_{k}$$

where $x_{k}$ is $u_{k}$ and represents the *k*-th mode component.

### 2.3. Multiscale Permutation Entropy

MPE is an effective feature extraction method based on the PE. The calculation process of this method is mainly divided into two steps [27]. Firstly, the multiscale coarse-granulation time series is established. Secondly, the permutation entropy of coarse-grained sequences at different scales is calculated. The specific calculation process is as follows:

1. The original time series is set as $X=\left\{x_{i},i=1,2,\ldots ,N\right\}$, and the new sequence $y_{j}^{\left(s\right)}$ is obtained by coarse granulation:
  (11) $$y_{j}^{\left(s\right)}=\frac{1}{s}\sum_{i=\left(j-1\right)s+1}^{js}x_{i},\;\;j=1,2,\ldots ,\left[N/S\right]$$
  where *s* represents the scale factor.
2. $Y_{l}^{s}$ is obtained by phase space reconstruction for each coarse-grained sequence $y_{j}^{\left(s\right)}$:
  (12) $$Y_{l}^{s}=\left\{y_{l}^{\left(s\right)},y_{l+\tau }^{\left(s\right)},\ldots ,y_{l+\left(m-1\right)\tau }^{\left(s\right)}\right\}$$
  where m represents the embedded dimension, and τ represents the delay time.
3. Arrange the reconstructed time series in ascending order. There are m! possible permutations for the phase space embedded in *m* dimensions. Count the number of possible occurrences of the *r*-th permutation, denoted as $N_{r}$, where r=1,2,…,R(R≤m!). Then, the probability of the occurrences of the *r*-th permutation is
  (13) $$P_{r}=\frac{N_{r}}{\frac{n}{s}-m+1}$$
4. Finally, the multiscale permutation entropy is obtained:
  (14) $$H_{p}=-\sum_{r=1}^{R}P_{r}\ln P_{r}$$

### 2.4. Particle Swarm Optimization-Based SVM

**Figure 1:**
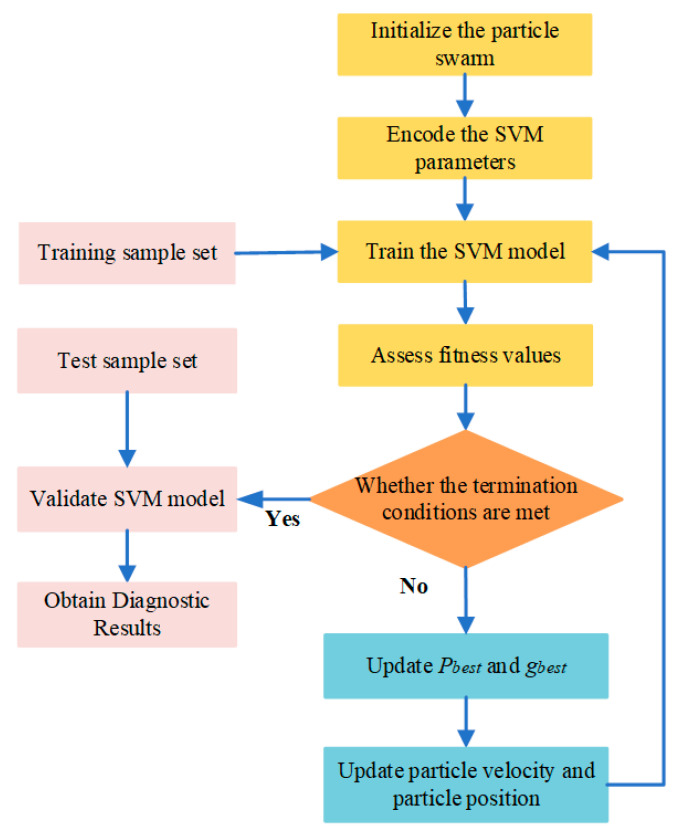
Algorithm flow chart of optimizing SVM parameters using PSO.

SVM can effectively deal with small sample, nonlinear and local minimum problems. In the SVM with the Gaussian kernel, the identification accuracy is closely related to the penalty parameter *c* and the nuclear parameter *g*. It is difficult to choose the optimal combination parameters *c* and *g* by virtue of expert experience. Therefore, some optimization algorithms are adopted to find the optimal combination parameters *c* and *g*. Common optimization algorithms include the genetic algorithm (GA) [28] and particle swarm optimization algorithm (PSO) [29,30]. Xue et al. [31] used the PSO-SVM diagnostic model to diagnose the tension of wire rope in the hoisting system and compared it with the GA-SVM diagnostic model. The results show that the identification accuracy of PSO-SVM is higher than GA-SVM. In addition, PSO has the characteristics of simple calculation, fast convergence speed and strong convergence ability. Therefore, this paper adopts the PSO algorithm to optimize the penalty parameter *c* and kernel parameter *g* of SVM. Figure 1 shows the algorithm flow of optimizing SVM parameters using PSO. The specific steps are as follows:

Step 1. Initialize the particle swarm. Initialize the particle swarm parameters, including particle number, learning factor, weighting coefficient, particle position and particle velocity.

Step 2. Encode the SVM parameters. The penalty parameter *c* and kernel parameter *g* of SVM are encoded as the position of the particle.

Step 3. Train the SVM model. The SVM model is trained with training samples. The parameters *c* and *g* vary as the particle travels.

Step 4. Assess fitness values. Use Equation (13) to calculate and evaluate the fitness value of particles. The fitness value is used to evaluate the validity of the fault diagnosis model with the combined parameters *c* and *g*. A larger fitness value indicates a higher fault diagnosis accuracy:

(15) $$f(pop^{i})=1-\frac{y_{i}}{y_{c}+y_{i}}$$

where $f(pop^{i})$ is the fitness used to determine the accuracy of the classifier, $y_{i}$ is the number of wrongly classified and $y_{c}$ is the number of correctly classified.

Step 5. Determine the stop conditions. When the desired accuracy is reached, the iteration is terminated, and the optimal combined parameters *c* and *g* of SVM are obtained. Otherwise, continue iterating.

Steps 6. Update the parameters. Update pbest and gbest. The particle velocity and particle position are updated according to Equations (14) and (15):

(16) $$v_{id}^{k}=\omega v_{id}^{k-1}+c_{1}r_{1}\left(pbest_{id}-x_{id}^{k-1}\right)+c_{2}r_{2}\left(gbest_{d}-x_{id}^{k-1}\right)$$

(17) $$x_{id}^{k}=x_{id}^{k-1}+v_{id}^{k-1}$$

where $v_{id}^{k}$ is the motion velocity of the *k*-th iteration particle, $x_{id}^{k}$ is the position of the *k*-th iteration particle in the current search space, $c_{1}$ and $c_{2}$ are acceleration factors, $r_{1}$ and $r_{2}$ are two random constants in the value range [0, 1], ω is the inertial weight, $pbest_{id}$ is the historical optimal position of a single particle and $gbest_{d}$ is the historical optimal position of the particle swarm.

## 3. Rolling Bearing Fault Diagnosis Based on VMD-MPE and PSO-SVM

**Figure 2:**
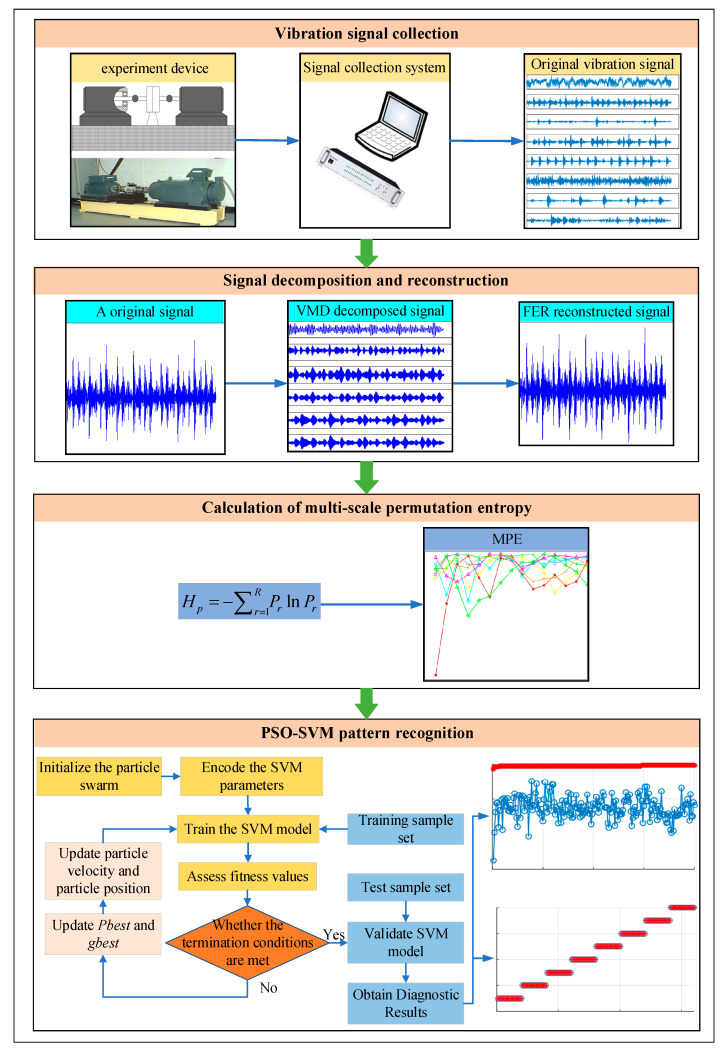
Flow chart of bearing fault diagnosis based on the proposed method.

To realize accurate diagnosis of bearing faults, firstly, VMD is used to decompose the vibration signal collected by the sensor, and FER is used to reconstruct the IMF component of VMD decomposition. Secondly, the MPE of the reconstructed signals is calculated to construct multidimensional feature vectors. Finally, PSO-SVM is used for bearing fault diagnosis. Figure 2 shows the rolling bearing fault diagnosis process based on VMD-MPE and PSO-SVM. The specific steps are as follows.

Step 1. Bearing vibration signal collection. The vibration signals of various fault states of rolling bearing are collected by using sensors.

Step 2. Signal decomposition and reconstruction. VMD is used to decompose bearing vibration signals into several IMF components, and FER is used to reconstruct the original bearing vibration signal, which can retain the useful fault characteristic information and remove some interference frequency components.

Step 3. Multiscale permutation entropy-based fault feature extraction. The multiscale permutation entropy of the reconstructed signals is calculated to construct multidimensional feature vectors.

Step 4. Pattern recognition based on PSO-SVM. The extracted multidimensional feature vector is randomly divided into the training sample set and testing sample set, where the training sample set is used for training the PSO-SVM model, and the testing sample set is input into the well-trained PSO-SVM model to identify different health conditions of bearings and automatically output the recognition results.

## 4. Experimental Verification

### 4.1. Case 1: Bearing Data from CWRU

#### 4.1.1. Data Collection and Sample Selection

**Figure 3:**
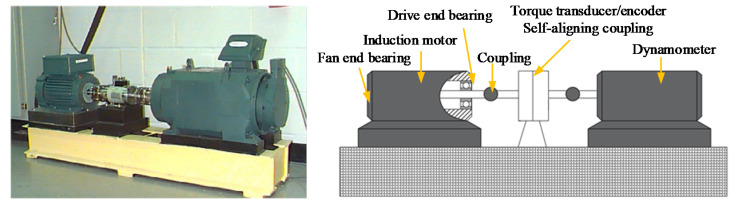
Rolling bearing fault simulation experimental device.

**Figure 4:**
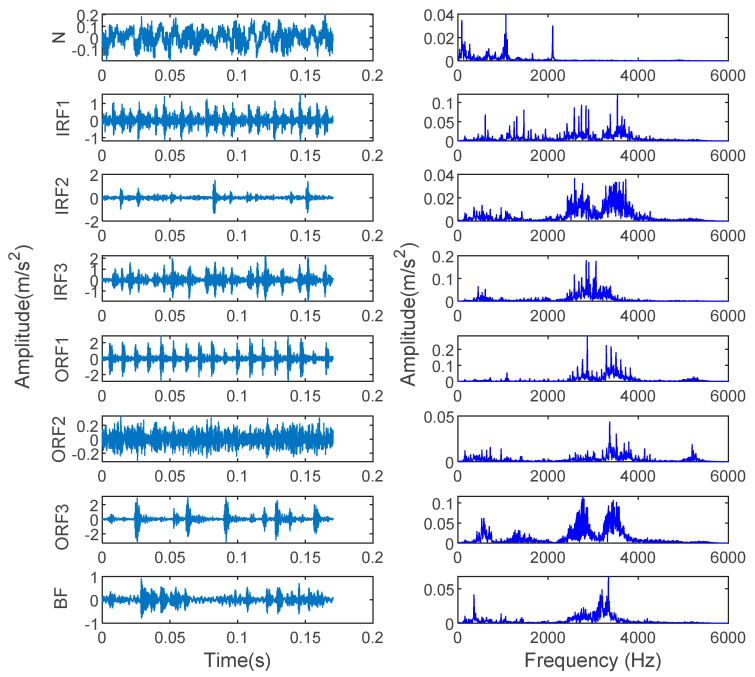
Time-domain waveform and spectrum of 8 bearing vibration signals in case 1.

The data used in the experiment came from the rolling bearing database provided by the Electrical Engineering Laboratory of Case Western Reserve University (CWRU). Figure 3 shows the rolling bearing experimental system. The adopted rolling bearing is SKF6205 in case 1. The single fault types consist of inner race fault (IRF), outer race fault (ORF) and ball fault (BF) with the fault diameters of 0.1778, 0.3556 and 0.5334 mm. These single faults are caused by the electrodischarge machine (EDM). The data used in this paper are under the condition that the motor load is 1 horsepower, the sampling frequency is 12 kHz, the sampling location and fault location are both at the driving end and the motor speed is 1772 r/min. Eight kinds of vibration signals collected at the driving end are regarded as experimental data in case 1. Fifty groups of samples are taken for each classification, and the length of each group is 2048 points. Thirty groups of data are randomly selected as a training sample set, and the rest are used as a test sample set. Table 1 shows the specific information of sample selection. Figure 4 shows the time-domain waveform and spectrum of vibration signals of eight types of bearings in case 1. Due to the similarity of the time-domain waveform and spectrum components, it is not easy to automatically identify the fault types and degrees of bearings through observing Figure 4. Therefore, an intelligent diagnosis algorithm is needed to realize automatic identification of bearing fault types and degrees.

#### 4.1.2. Fault Diagnosis Results and Comparative Analysis

**Figure 5:**
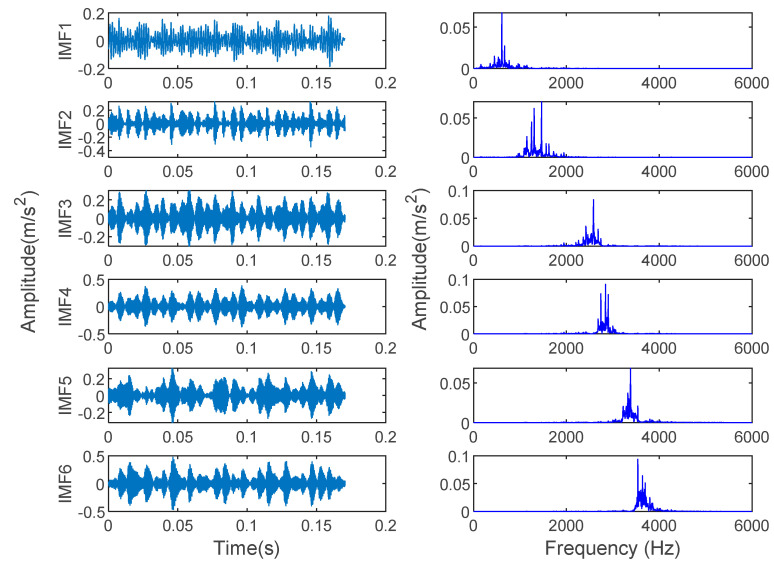
Time-domain waveform and spectrum of IMF component obtained using VMD for inner race fault signal in case 1.

**Figure 6:**
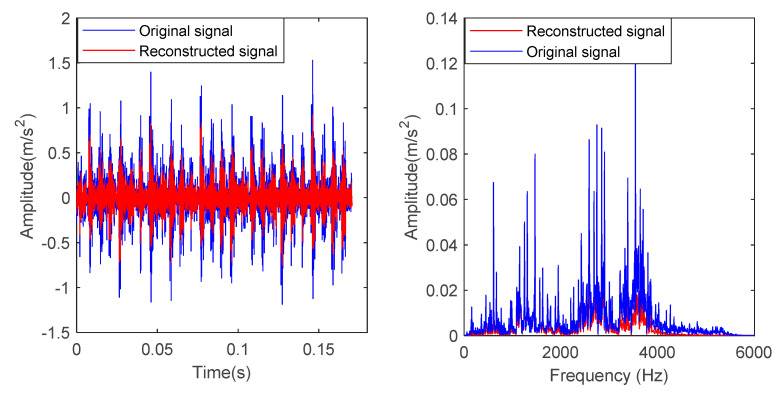
Time-domain waveform and spectrum of the inner race fault signal before and after reconstruction in case 1.

**Figure 7:**
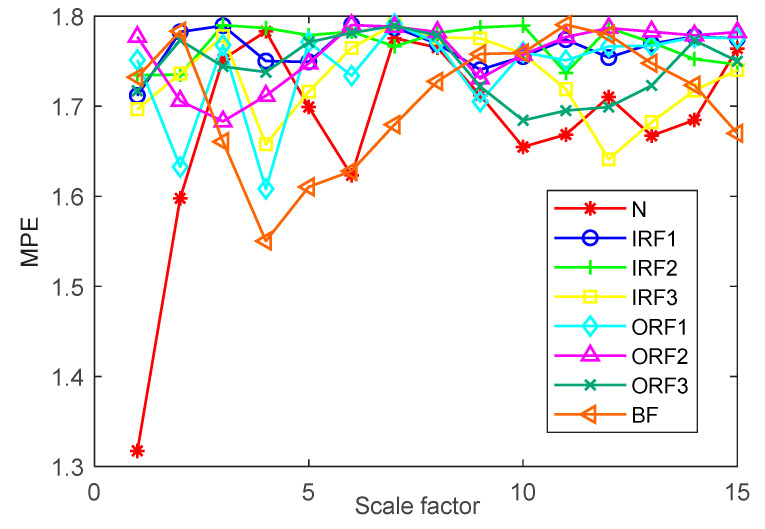
Multiscale permutation entropy at different scales in case 1.

**Figure 8:**
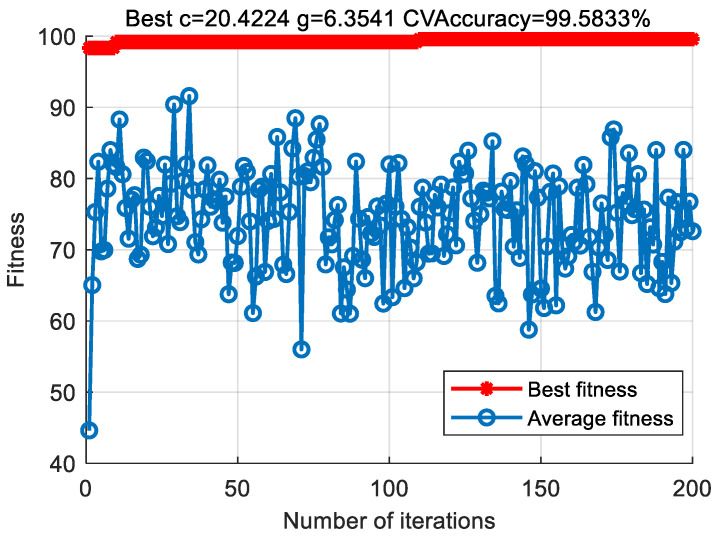
Optimization results of SVM parameters using PSO in case 1.

**Figure 9:**
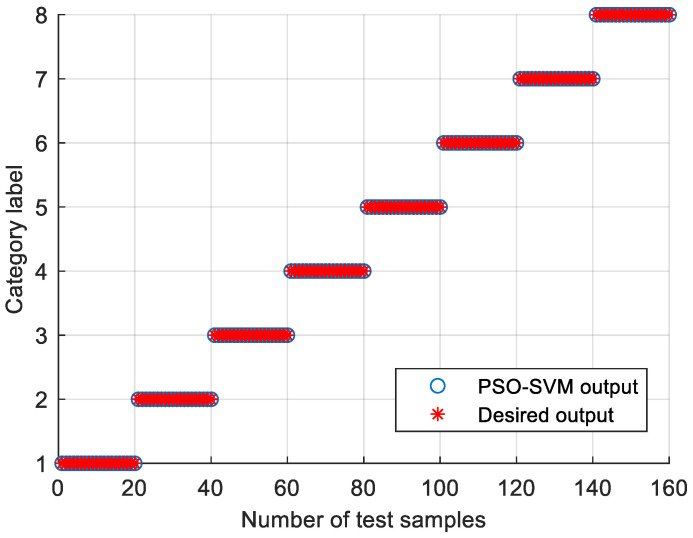
Identification results of the proposed method in case 1.

In order to verify the effectiveness of the proposed method, the bearing vibration data of case 1 are analyzed by using the proposed method. First, according to the algorithm flow chart shown in Figure 2, the original bearing vibration signal of case 1 is decomposed into several IMF by using the VMD method. Take the bearing inner race fault (IRF1) signals as an example. Figure 5 shows the VMD decomposition results of bearing inner race fault signals. Then, the decomposed component of VMD is reconstructed. The feature energy ratio $FER_{k}$ of each IMF component is calculated according to Equation (6). The bearing inner race fault feature frequency in case 1 is 159.92 Hz. Theoretically, the higher the order frequency is, the higher the recognition accuracy will be. However, in practical application, the fault feature information is mainly concentrated in the first few order frequencies. The higher the order frequency, the lower the operating efficiency. Considered comprehensively, the first three order frequencies are selected for calculation in this paper. Then, according to Equations (8) and (9), the reconstruction weight $\beta_{k}$ of each component and the normalized reconstruction weight ${\hat{\beta }}_{k}$ are calculated. Finally, reconstructed signal $x_{Final}$ is obtained according to Equation (10). Table 2 shows the calculation results of $FER_{k}$, $\beta_{k}$ and ${\hat{\beta }}_{k}$. Figure 6 shows the time-domain waveform and spectrum of the reconstructed signal. Then, the MPE of the reconstructed signals is calculated and a multidimensional eigenvector is established. Figure 7 shows the MPE at different scales. Finally, the extracted multidimensional feature vectors are input into PSO-SVM for automatic fault classification. Figure 8 shows the optimization results of SVM parameters using PSO. It can be seen from Figure 8 that the optimal combination parameters of SVM determined by the PSO algorithm are *c* = 20.42 and *g* = 6.35, and the accuracy of cross-validation is 99.58%. Figure 9 shows the classification results of the proposed method. As can be seen from Figure 9, the fault identification accuracy of the proposed method is as high as 100%. The effectiveness of the proposed method in the classification and degree identification of bearing faults is preliminarily demonstrated. In addition, the validity of the proposed method is further verified from the following six angles.

**Figure 10:**
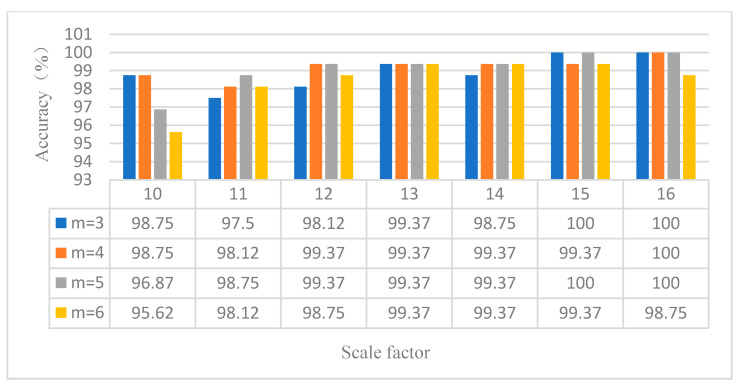
Identification results of the proposed method under different embedding dimensions and scale factors in case 1.

**Figure 11:**
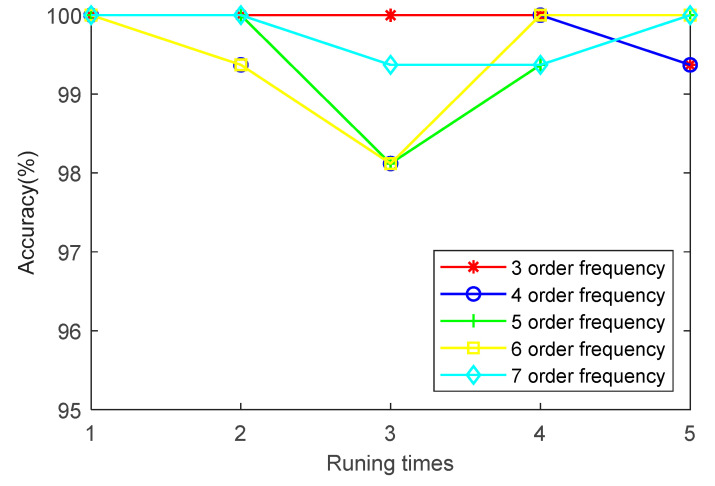
Identification results of five trials of the proposed method at different order frequencies in case 1.

(1) The influence of the embedding dimension and scale factor on the diagnosis results of the proposed method is investigated. MPE depends on the embedding dimension m and scale factor s. When m is too large, the calculation efficiency of MPE is slow. When m is too small, small changes in time series cannot be detected [32]. According to literature [33], it can be seen that generally, m is between 3 and 7, and s is greater than 10. Figure 10 shows the identification results of the proposed method under different embedding dimensions and scale factors. It can be seen from Figure 10 that when the embedding dimension *m* = 3 and the scale factor *s* = 15, the proposed method achieves the highest identification accuracy. The validity of the parameter selection of the proposed method is verified in case 1.

(2) The influence of different training sample ratios on the identification results of rolling bearing fault states is analyzed. This article selects a total of 400 samples of the above eight types of data. Randomly take 20%, 30%, 40%, 50% and 60% samples of each category as the training set and the rest as the test set. Table 3 shows the identification results under different training samples. Seen from Table 3, with the increase of the number of training samples, the identification accuracy of the proposed method also increases. When the training samples reach 60% of the total samples, that is, the number of training samples in each classification is 30, the fault identification accuracy of the proposed method in this paper can reach 100%. In case 1, this proves the effectiveness and feasibility of the number of training samples selected in the proposed method.

(3) To verify the effectiveness of the proposed method using VMD and MPE, the proposed method (VMD-MPE-PSOSVM) and some similar available methods (e.g., EMD-MPE-PSOSVM, WT-MPE-PSOSVM, VMD-MSE-PSOSVM, VMD-MFE-PSOSVM) are used to analyze the abovementioned same experimental data. Table 4 shows the identification results of the five methods. It can be seen from Table 4 that the average identification accuracy of the proposed method is 99.87%, which is significantly higher than the identification accuracy of the other four methods. It can also be seen from Table 4 that the standard deviation of the proposed method is the smallest compared with other methods, which verifies that the proposed method runs stably. This fully verifies the effectiveness of using VMD and MPE in the proposed method in case 1.

(4) To illustrate the superiority of using PSO to optimize the important parameters of the SVM model used in the proposed method, VMD-MPE-PSOSVM and VMD-MPE-SVM are adopted to analyze the same bearing experimental data. Table 5 lists the comparative results of the two methods. It is obvious from Table 5 that the choice of parameters *c* and *g* has a great influence on the classification results. The randomly selected parameters *c* and *g* cannot guarantee that the classification accuracy of SVM achieves the desired effect, but the combination parameters (i.e., *c* and *g*) of SVM in the proposed method are determined automatically by applying the PSO method, which can achieve a higher identification accuracy. This further verifies the superiority of using PSO to optimize SVM parameters in the proposed method in case 1.

(5) To investigate the effect of order frequency on the identification accuracy of the proposed method, the first three, four, five, six and seven order frequencies are employed to analyze the bearing data of case 1. Figure 11 shows the identification results of running five times at different order frequencies. It can be seen from Figure 11 that the order frequency has little effect on the identification accuracy. The identification accuracy of case 1 is mainly between 98% and 100%. It is demonstrated that the selection of order frequency of the proposed method is appropriate in case 1.

(6) To discuss the effect of the VMD method, VMD-MPE-PSOSVM and MPE-PSOSVM are used to analyze the same bearing experimental data. Table 6 gives the comparative results of the two methods. It can be seen from Table 6 that the identification accuracy with the VMD method is significantly higher than that without the VMD method. It is demonstrated that the VMD method has advantages in the signal decomposition process and can improve the fault identification accuracy.

### 4.2. Case 2: Bearing Data from Laboratory

#### 4.2.1. Data Collection and Sample Selection

**Figure 12:**
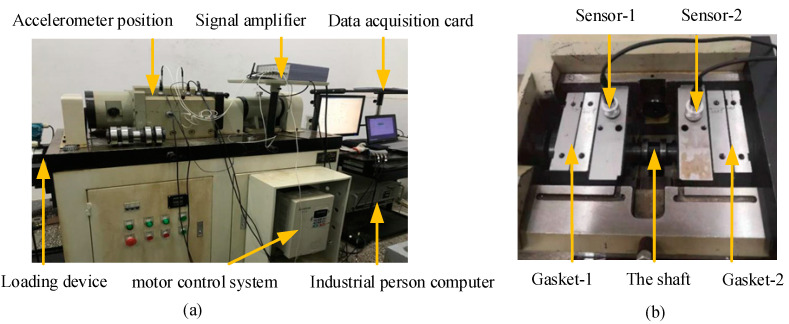
Experimental apparatus for case 2.

**Figure 13:**
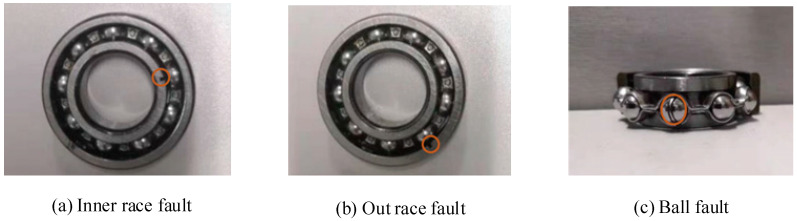
Three kinds of rolling bearing faults.

**Figure 14:**
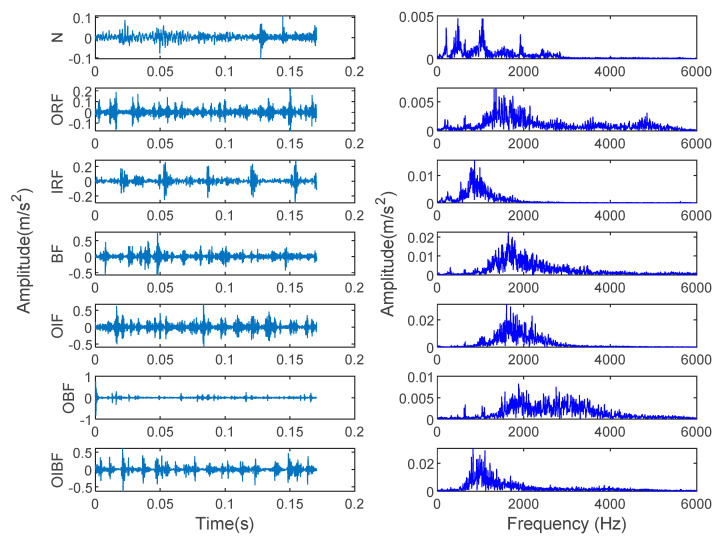
Time-domain waveform and spectrum of 7 bearing vibration signals in case 2.

The data used in case 2 come from the author’s laboratory. ABLT-1A as the experimental device is shown in Figure 12a, which is mainly composed of a computer control system, a test head (as shown in Figure 12b), a lubrication system, a transmission system, a loading system, a motor control system and a data acquisition system. The motor speed is adjusted by the motor control system. The motor drives the shaft to rotate, and the fault bearing is installed on the shaft. The corresponding parameters of the rolling bearing used in case 2 are shown in Table 7. The whole data set includes seven fault states of the rolling bearing: normal (N), outer race fault (ORF), inner race fault (IRF), ball fault (BF), outer-inner races compound fault (OIF), outer race and ball compound fault (OBF) and outer-inner races and ball compound fault (OIBF). These faults are caused by the electrodischarge machine (EDM). Figure 13 shows the three faults in this case. The flaw size of inner race, outer race and ball is 1 mm in width. The NI9234 data acquisition card and two PCB accelerometers (i.e., sensor-1 and sensor-2) are adopted to collect the bearing vibration data, where one PCB accelerometer (sensor-2) is moved and mounted at a certain distance from the faulty bearing to simulate the weak fault signal. The motor load is 5.1 kN. The rotating speed and sampling frequency set as 1050 r/min and 12 kHz, respectively. Fifty groups of samples collected in sensor-2 are selected for each health condition, where 30 groups of data samples are randomly selected as the training sample set, and the rest are used as the test sample set. Note that the length of each group of samples is 2048 points. Table 8 shows the specific information of sample selection. Figure 14 shows the time-domain waveform and frequency spectra of seven bearing vibration signals. It can be seen from Figure 14 that the fault type of the bearing cannot be identified by observing the time-domain waveform and frequency spectrum. Therefore, it is necessary to employ an intelligent diagnosis algorithm to automatically identify bearing fault types.

#### 4.2.2. Fault Diagnosis Results and Comparative Analysis

**Figure 15:**
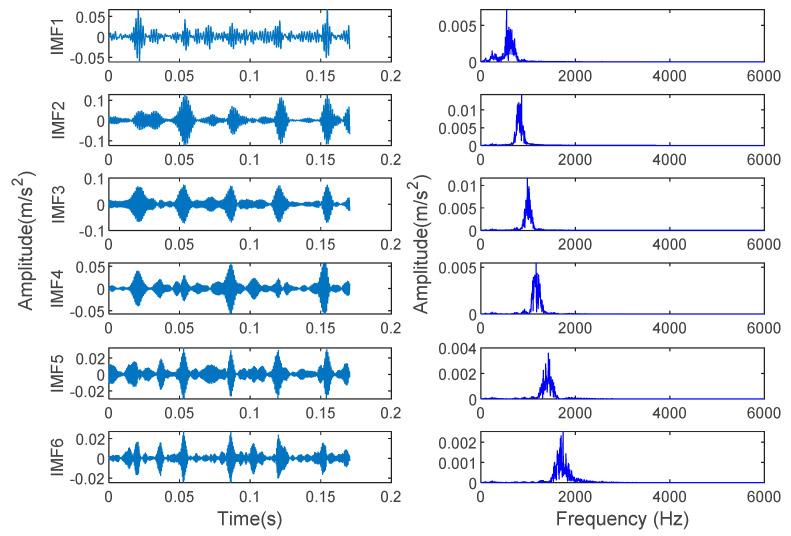
Time-domain waveform and spectrum of IMF components obtained using VMD for inner race fault signal in case 2.

**Figure 16:**
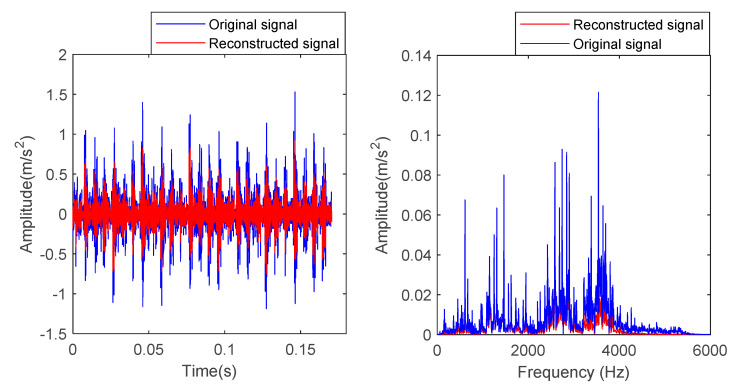
Time-domain waveform and spectrum of the inner race fault signal before and after reconstruction in case 2.

**Figure 17:**
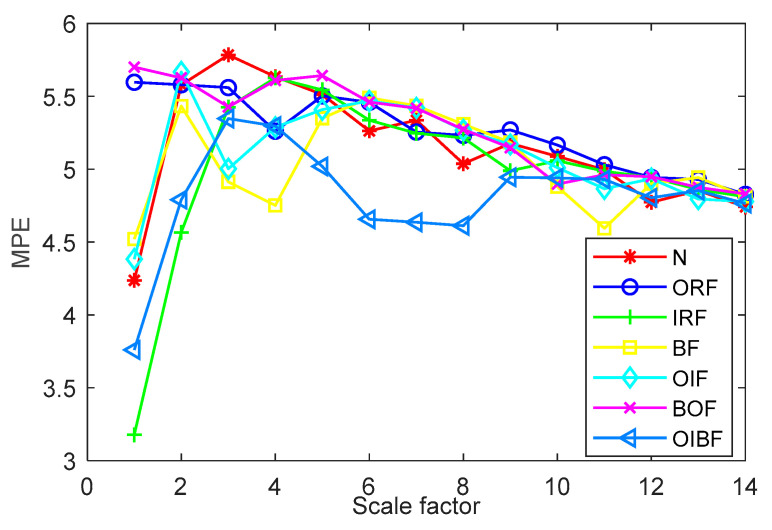
Multiscale permutation entropy at different scales in case 2.

**Figure 18:**
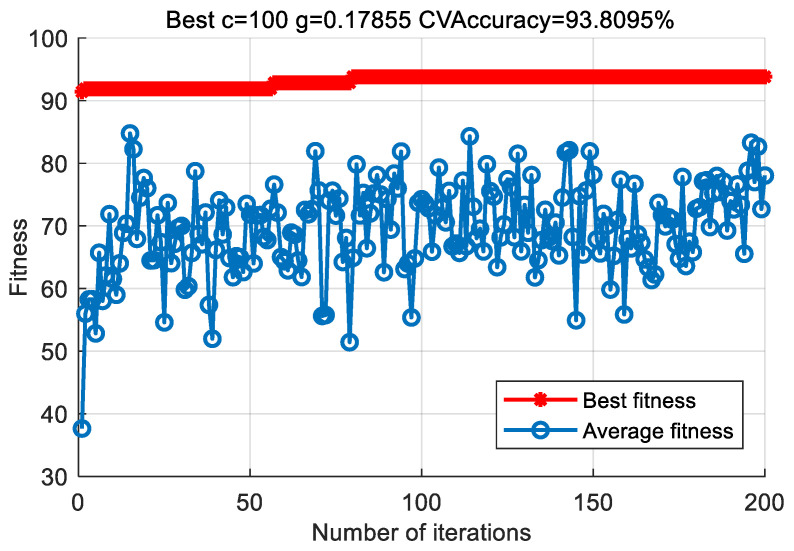
Optimization results of SVM parameters using PSO in case 2.

**Figure 19:**
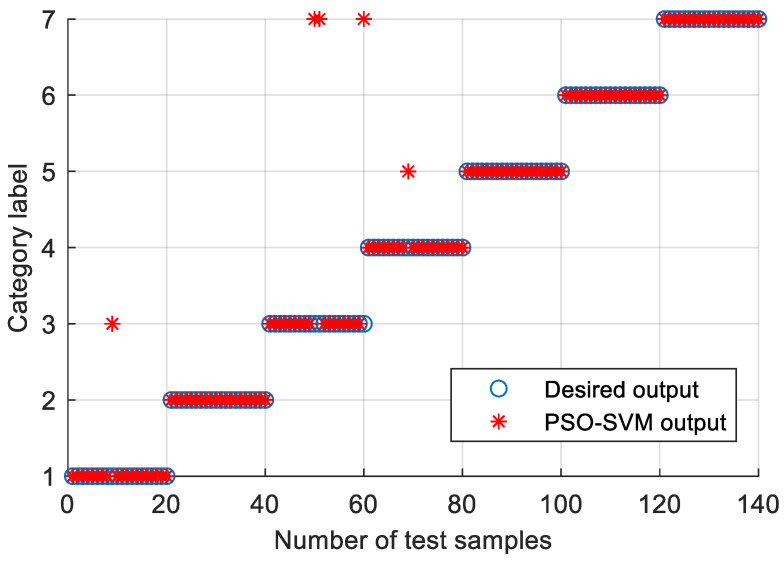
Identification results of the proposed method in case 2.

In order to prove the superiority of the proposed method, the proposed method in this paper is used to analyze the bearing vibration data of case 2. First, according to the algorithm flow chart shown in Figure 2, the original bearing vibration signal of case 2 is decomposed into several IMF by using the VMD method. Take the case 2 bearing inner ring fault (IRF) signal as an example. Figure 15 shows the VMD decomposition result of the fault signal of the bearing inner ring in case 2. Subsequently, the reconstruction method based on the FER criterion is used to reconstruct the IMF components. The feature energy ratio $FER_{k}$ of each IMF component is calculated according to Equation (6). The feature frequency of the inner race fault in case 2 is 94.76 Hz. Theoretically, the higher the order frequency is, the higher the recognition accuracy will be. However, in practical application, the fault feature information is mainly concentrated in the first few order frequencies. The higher the order frequency, the lower the operating efficiency. Considered comprehensively, the first three order frequencies are selected for calculation in this paper. Then, the reconstruction weight $\beta_{k}$ of each component and the normalized reconstruction weight ${\hat{\beta }}_{k}$ are calculated according to Equations (8) and (9). Finally, the reconstructed signal $x_{Final}$ is obtained from Equation (10). Table 9 shows the calculation results of $FER_{k}$, $\beta_{k}$ and ${\hat{\beta }}_{k}$. Figure 16 shows the result of the reconstructed signal. Then, the MPE of the reconstructed signal is calculated to form a multidimensional feature vector. Figure 17 shows the MPE at different scales. Finally, the extracted multidimensional feature vectors are input into PSO-SVM for pattern identification. Figure 18 shows the optimization results of SVM parameters using PSO. It can be seen from Figure 18 that the optimal combination parameters of the SVM by the PSO algorithm are *c* = 100 and *g* = 0.17, and the cross-validation accuracy rate is 93.80%. Figure 19 shows the classification results of the proposed method. It can be seen from Figure 19 that the proposed method can achieve a fault identification rate of 96.42%. This is mainly because the vibration data of single faults and compound faults are collected in case 2. Moreover, their fault feature information is not obvious. Concretely, compared with case 1, the difference of the time-domain waveform of each bearing fault type is relatively small in case 2. That is, in case 2, it is more difficult to identify bearing fault patterns by directly observing the time-domain waveform of the bearing vibration signal, which indicates that the identification accuracy of the bearing fault may be reduced (i.e., less than 100%) by using the proposed method to analyze the bearing vibration data of case 2. The effectiveness of the proposed method in identifying bearing fault types is preliminarily verified. In addition, the validity of the proposed method is further verified from the following six angles.

**Figure 20:**
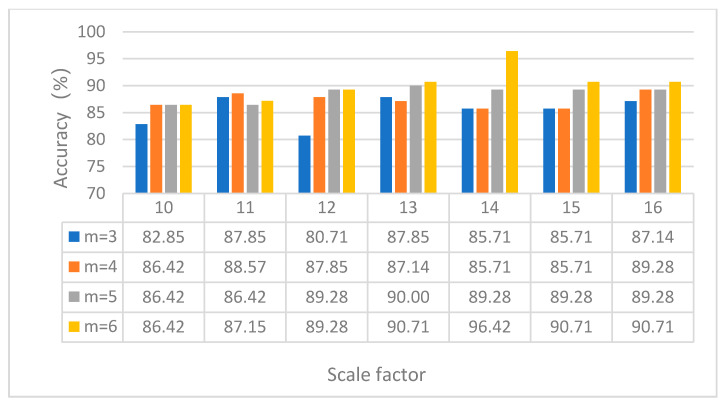
Identification results of the proposed method under different embedding dimensions and scale factors in case 2.

**Figure 21:**
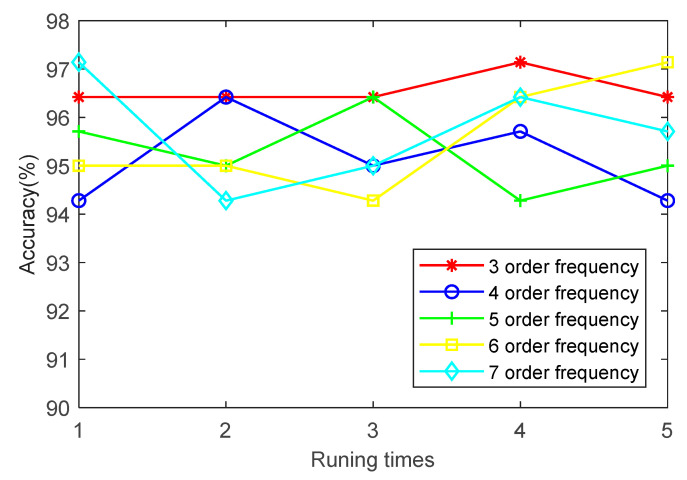
Identification results of five trials of the proposed method at different order frequencies in case 2.

(1) The influence of the embedding dimension and scale factor on the diagnosis result of the proposed method is analyzed. Figure 20 shows the identification results of the proposed method under different embedding dimensions and scale factors. It can be seen from Figure 20 that when the embedding dimension *m* = 6 and the scale factor *s* = 14, the proposed method achieves the highest identification accuracy. The validity of the parameter selection of the proposed method is verified in case 2.

(2) In order to study the influence of different training sample on the accuracy of rolling bearing fault state identification, 350 samples of case 2 data are selected for analysis. Randomly take 20%, 30%, 40%, 50% and 60% samples of each category as the training sample set, and the rest as the test sample set. Table 10 shows the identification results under different training samples. It can be observed from Table 10 that when the number of training samples increases, the identification accuracy also increases. When the number of training samples reaches 60% of the total samples, the number of training samples for each classification is 30, and the fault identification accuracy of the method proposed in this paper can reach 96.42%. This proves the effectiveness of selecting the number of training samples of the proposed method in case 2.

(3) In order to examine the effects of using VMD and MPE in the proposed method, five methods (e.g., VMD-MPE-PSOSVM, VMD-MSE-PSOSVM, VMD-MFE-PSOSVM, EMD-MPE-PSOSVM and WT-MPE-PSOSVM) are adopted to compare the same comparative analysis of the bearing experimental data. Table 11 shows the diagnosis results of the different methods. It can be clearly observed from Table 11 that the proposed method has the highest average identification accuracy (96.56%) compared with the other methods. It can also be seen from Table 9 that the standard deviation of the proposed method is the smallest compared with other methods, which verifies that the proposed method runs stably. This further proves the superiority of combining VMD and MPE of the proposed method in bearing health condition identification.

(4) To verify the effectiveness of using PSO to optimize SVM parameters in this method, VMD-MPE-PSOSVM and VMD-MPE-SVM are used to analyze the same bearing experimental data. Table 12 lists the comparison results of the two methods. It is obvious from Table 12 that the parameters *c* and *g* selected by experience cannot be guaranteed to achieve the highest classification accuracy of SVM, but the combination parameters (i.e., *c* and *g*) of SVM are obtained by utilizing the PSO method, which can assure identification accuracy is the highest. This fully verifies the effectiveness of employing PSO to optimize SVM parameters in the proposed method in case 2.

(5) To study the effect of order frequency on the identification accuracy of the proposed method, the first three, four, five, six and seven order frequencies are employed to analyze the bearing data of case 2. Figure 21 shows the identification results of running 5 times at different order frequencies. It can be seen from Figure 21 that with the increase of order frequency, the identification accuracy has no obvious change. The identification accuracy of case 2 is between 94% and 97%. It is verified that the selection of the order frequency of the proposed method is reliable in case 2.

(6) To discuss the effect of the VMD method, VMD-MPE-PSOSVM and MPE-PSOSVM are used to analyze the same bearing experimental data. Table 13 gives the comparative results of the two methods. It can be seen from Table 13 that the identification accuracy with the VMD method is significantly higher than that without the VMD method. It is demonstrated that the VMD method has advantages in the signal decomposition process and can improve the fault identification accuracy.

### 4.3. Further Discusses

Through the comparison and analysis of different methods, it is concluded that the proposed method combines the advantages of VMD, MPE and SVM, which can effectively improve the identification accuracy of rolling bearings. It is fully proven that the proposed method has a certain application value for bearing fault diagnosis. Our future research directions are summarized as the following four points.

Firstly, although the identification accuracy of the proposed method is relatively high, some key parameters (e.g., the embedding dimension *m* and scale factor *s* of MPE) have a great impact on the identification results. Therefore, in our future research, we will adopt some appropriate optimization algorithms (e.g., grasshopper optimization algorithm (GOA), grey wolf optimization algorithm (GWO) and bat algorithm (BA)) to find the optimal parameters of MPE.

Secondly, in this paper, bearing health status under constant speed is analyzed. However, the diagnostic performance of the proposed method may also be affected when the rotational speed and load are dynamically changing. Hence, our next study will investigate how to use the proposed method to solve this problem.

Thirdly, for a small number of samples, SVM has a prominent diagnostic effect, but the identification performance of SVM may be reduced when it is faced with a big data scenario. Therefore, in future research, we will use deep learning (DL) [34,35] instead of SVM to identify bearing fault patterns.

Finally, from the prospect of application, the method proposed in this paper will be further studied and extended to detect more mechanical equipment faults (e.g., wind turbine generator, locomotive fault detection) in future work.

## 5. Conclusions

This paper proposes a bearing fault diagnosis method based on variational mode decomposition (VMD), multiscale permutation entropy (MPE) and the particle swarm optimization-based support vector machine (PSO-SVM). Experiments and comparative analysis verify the effectiveness and superiority of the proposed method. The main work and innovation of this paper are summarized as follows:

(1) The variational mode decomposition method based on the feature energy ratio (FER) criterion is applied to decompose and reconstruct the original bearing vibration signal, which can retain the useful fault characteristic information and remove some interference frequency components.

(2) The particle swarm optimization algorithm is adopted to optimize the combination parameters of the support vector machine, which can reduce the influence of manual parameters on the classification performance and improve the generalization performance of the support vector machine. Compared with the support vector machine, the particle swarm optimization-based support vector machine has obvious superiority in identifying bearing fault patterns.

(3) The analysis results of two experimental examples show that the identification accuracy of the proposed method can achieve, respectively, 100% and 96.42% in identifying bearing fault categories and severities. Furthermore, compared with some similar diagnostic methods (e.g., EMD-MPE-PSOSVM, WT-MPE-PSOSVM, VMD-MSE-PSOSVM and VMD-MFE-PSOSVM), the proposed method can achieve a higher identification accuracy, which proves the validity of the proposed method in bearing health condition identification.

## Figures and Tables

**Table 1:** Sample information of rolling bearing data in case 1.

Fault Type	Fault Size/mm	Number of Training Samples	Number of Test Samples	Category Label
Normal (N)	0	30	20	1
IRF1	0.1778	30	20	2
IRF2	0.3556	30	20	3
IRF3	0.5334	30	20	4
ORF1	0.1778	30	20	5
ORF2	0.3556	30	20	6
ORF3	0.5334	30	20	7
BF	0.3556	30	20	8

**Table 2:** Calculation results of $FER_{k}$, $\beta_{k}$ and ${\hat{\beta }}_{k}$ in case 1.

IMF	$FER_{k}$	$\beta_{k}$	${\hat{\beta }}_{k}$
1	0.1320	0.1150	0.4243
2	0.3111	0.2711	1
3	0.2098	0.1828	0.6742
4	0.2395	0.2087	0.7698
5	0.1050	0.0915	0.3377
6	0.1497	0.1304	0.4811

**Table 3:** Identification results of the proposed method under different training sample ratios in case 1.

**Training Samples Ratio**	20%	30%	40%	50%	60%
**Identification Accuracy (%)**	93.75	95.00	96.25	98.50	100

**Table 4:** Identification results of different feature extraction methods with PSO-SVM in case 1.

Feature Extraction Method	Identification Accuracy (%)					Average Identification Accuracy (%)	Standard Deviation
	1	2	3	4	5		
VMD-MPE	100	100	100	100	99.37	99.87	0.06
EMD-MPE	98.75	98.12	98.75	98.75	98.12	98.49	0.10
WT-MPE	41.25	41.25	41.87	41.25	41.87	41.64	0.11
VMD-MSE	93.75	92.50	94.37	92.50	92.50	93.12	0.62
VMD-MFE	93.75	94.37	94.37	95.00	95.62	94.62	0.41

**Table 5:** Identification accuracy of the proposed method with optimized and unoptimized parameters in case 1.

Classifier	Penalty Parameter *c*	Nuclear Parameter *g*	Identification Accuracy (%)
PSO-SVM	20.42	6.35	100
SVM	4.36	10	96.25
	22	70.17	62.50
	48.25	51.36	70.62
	90	35	80.62
	30	100	51.87

**Table 6:** Identification accuracy with VMD method and without VMD method in case 1.

Fault Diagnosis Method	Identification Accuracy (%)					Average Identification Accuracy (%)
	1	2	3	4	5	
VMD-MPE-PSOSVM	100	100	100	100	99.37	99.87
MPE-PSOSVM	93.75	90.62	95.00	92.50	95.00	93.37

**Table 7:** The corresponding parameters of rolling bearing.

Bearing Type	Inside Diameter	Roll Diameter	Outside Diameter	Number of the Roller	Contact Angle (°)
HRB 6205	25 mm	7.94 mm	52 mm	9	0

**Table 8:** Sample information of rolling bearing data in case 2.

The Fault Types	Number of Training Samples	Number of Test Samples	Category Label
Normal (N)	30	20	1
ORF	30	20	2
IRF	30	20	3
BF	30	20	4
OIF	30	20	5
OBF	30	20	6
OIBF	30	20	7

**Table 9:** Calculation results of $FER_{k}$, $\beta_{k}$ and ${\hat{\beta }}_{k}$ in case 2.

IMF	$FER_{k}$	$\beta_{k}$	${\hat{\beta }}_{k}$
1	0.0146	0.2139	0.6691
2	0.0094	0.1385	0.4332
3	0.0078	0.1150	0.3597
4	0.0219	0.3198	1.0000
5	0.0051	0.0739	0.2311
6	0.0095	0.1386	0.4336

**Table 10:** Identification results of the proposed method under different training sample ratios in case 2.

**Training Sample Ratio**	20%	30%	40%	50%	60%
**Identification Accuracy (%)**	79.64	86.50	90.00	89.14	96.42

**Table 11:** Identification results of different feature extraction methods with PSO-SVM in case 2.

Feature Extraction Method	Identification Accuracy (%)					Average Identification Accuracy (%)	Standard Deviation
	1	2	3	4	5		
VMD-MPE	96.42	96.42	96.42	97.14	96.42	96.56	0.08
EMD-MPE	32.85	32.85	28.57	32.85	32.85	31.99	2.93
WT-MPE	88.57	90.00	90.00	88.57	87.87	89.00	0.73
VMD-MSE	85.70	84.28	82.14	82.14	84.28	83.71	1.91
VMD-MFE	80.71	82.14	80.71	81.42	80.71	81.14	0.33

**Table 12:** Identification accuracy of the proposed method with optimized and unoptimized parameters in case 2.

Classifier	Penalty Parameter *c*	Nuclear Parameter *g*	Identification Accuracy (%)
PSO-SVM	100	0.17	96.42
SVM	31.23	15.46	77.87
	58	5	92.14
	42	49.87	48.57
	89	30	63.57
	98.52	81	35.00

**Table 13:** Identification accuracy with VMD method and without VMD method in case 2.

Fault Diagnosis Method	Identification Accuracy (%)					Average Identification Accuracy (%)
	1	2	3	4	5	
VMD-MPE-PSOSVM	96.42	96.42	96.42	97.14	96.42	96.56
MPE-PSOSVM	90.00	91.42	89.28	91.42	89.28	90.28

## Data Availability

Data sharing not applicable.

## Nomenclature

VMD	Variational mode decomposition
WT	Wavelet transform
FER	Feature energy ratio
EMD	Empirical mode decomposition
SVM	Support vector machine
NB	Naive bayes
MSE	Multiscale sample entropy
IMF	Intrinsic mode functions
EEMD	Ensemble empirical mode decomposition
PE	Permutation entropy
DL	Deep learning
ANN	Artificial neural network
ELM	Extreme learning machine
WOA	Whale optimization algorithm
PSO	Particle swarm optimization
MFE	Multiscale fuzzy entropy
GA	Genetic algorithm
MPE	Multiscale permutation entropy
MFCNN	Multichannel fusion convolutional neural network
HMDSOF	Self-organizing fuzzy classifier based on harmonic mean difference
LMFO	Moth-flame optimization algorithm based on Lévy flight
GCMSDE	Generalized compound multiscale symbolic dynamic entropy
